# MARTin—an open-source platform for microarray analysis

**DOI:** 10.3389/fbinf.2024.1329062

**Published:** 2024-02-09

**Authors:** Kai O. Kreissner, Benjamin Faller, Ivan Talucci, Hans M. Maric

**Affiliations:** ^1^ Rudolf Virchow Center for Integrative and Translational Bioimaging, University of Würzburg, Würzburg, Germany; ^2^ Independent Scholar, Constance, Germany; ^3^ Department of Neurology, University Hospital Würzburg, Würzburg, Bavaria, Germany

**Keywords:** microarray, proteomics, array analysis, open source, image analysis, ImageJ, Java

## Abstract

**Background:** Microarray technology has brought significant advancements to high-throughput analysis, particularly in the comprehensive study of biomolecular interactions involving proteins, peptides, and antibodies, as well as in the fields of gene expression and genotyping. With the ever-increasing volume and intricacy of microarray data, an accurate, reliable and reproducible analysis is essential. Furthermore, there is a high level of variation in the format of microarrays. This not only holds true between different sample types but is also due to differences in the hardware used during the production of the arrays, as well as the personal preferences of the individual users. Therefore, there is a need for transparent, broadly applicable and user-friendly image quantification techniques to extract meaningful information from these complex datasets, while also addressing the challenges posed by specific microarray and imager formats, which can flaw analysis and interpretation.

**Results:** Here we introduce MicroArray Rastering Tool (MARTin), as a versatile tool developed primarily for the analysis of protein and peptide microarrays. Our software provides state-of-the-art methodologies, offering researchers a comprehensive tool for microarray image quantification. MARTin is independent of the microarray platform used and supports various configurations including high-density formats and printed arrays with significant *x* and *y* offsets. This is made possible by granting the user the ability to freely customize parts of the application to their specific microarray format. Thanks to built-in features like adaptive filtering and autofit, measurements can be done very efficiently and are highly reproducible. Furthermore, our tool integrates metadata management and integrity check features, providing a straightforward quality control method, along with a ready-to-use interface for in-depth data analysis. This not only promotes good scientific practice in the field of microarray analysis but also enhances the ability to explore and examine the generated data.

**Conclusion:** MARTin has been developed to empower its users with a reliable, efficient, and intuitive tool for peptidomic and proteomic array analysis, thereby facilitating data-driven discovery across disciplines. Our software is an open-source project freely available via the GNU Affero General Public License licence on GitHub.

## 1 Background

Microarrays in the form of miniaturized protein, DNA or peptide libraries have become an essential biotechnology, as they allow for rapid and efficient analysis of biomolecular interactions across disciplines. When produced under standardized conditions microarrays enable the screening and analysis in the context of genomic (Plomin and Schalkwyk, 2007; Akira et al., 2018), proteomic (Robinson et al., 2002; Schulte et al., 2023) and clinical (Talucci and Maric, 2023) research with the highest throughput and reproducibility. Numerous software tools have been developed to interpret array images and generate data from array experiments. Commercial options for the quantification of peptide-microarrays include PepSlide (SICASYS), ImaGene (Arrayit) and MAPIX (INNOPSYS). Free alternatives are plugins for open-source software like ImageJ or Bioconductor as well as standalone software alternatives like Array Analyze (Active Motif). Currently available solutions, which were highly specific to particular microarray formats, not only lacked adaptability beyond those formats but also suffered from inefficiencies in the measurement process, compromising their overall effectiveness and limiting their applicability. The reliable and reproducible data extraction after imaging analysis remains, however, often a challenge since available solutions commonly do not allow for adjustment for print-offsets, distortions and background variation, common and often unavoidable interferences from array production and processing. In addition, a versatile solution for custom printing formats or data acquisition array types is entirely missing. Beyond the quantification of microarray data, a multitude of analysis algorithms has been devised to automatically derive results through data aggregation (Renard et al., 2011; Parker Cates et al., 2021; Cathryn et al., 2022). To make algorithms such as these viable for mass data processing a certain degree of output-data standardization has to be met. Furthermore, the recording of relevant metadata would allow for additional data-analyses as well as quality control measures. As such there are many needs and wants potential microarray quantification software has to live up to.

**FIGURE 1:**
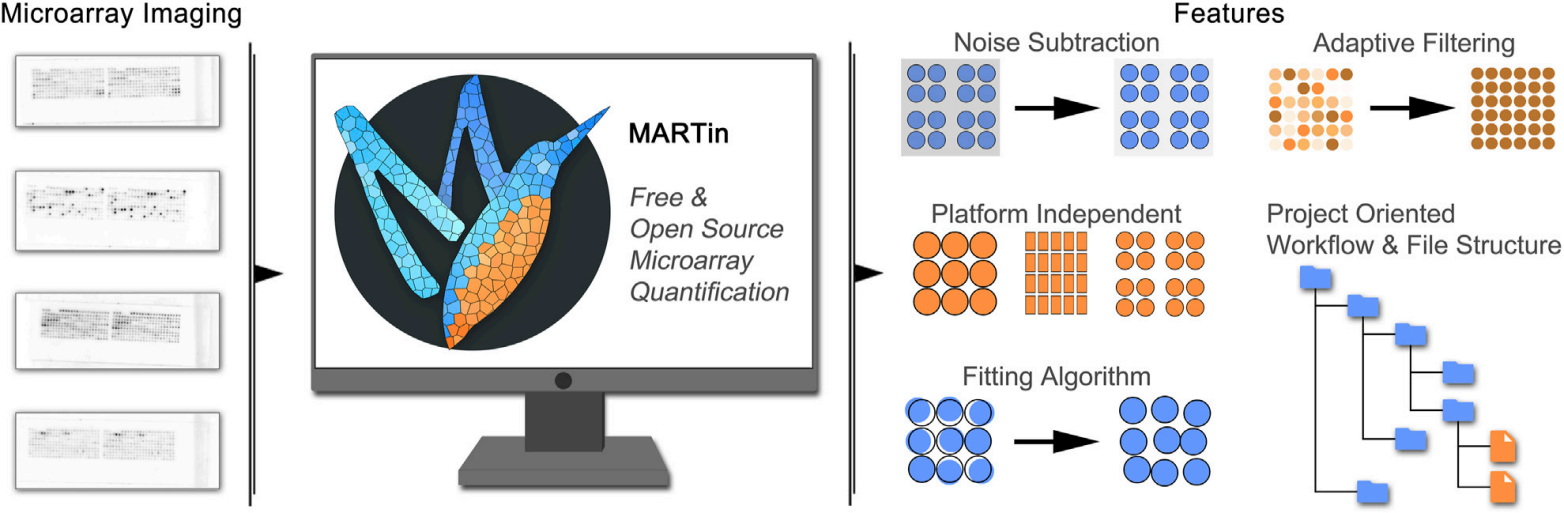
Use and features of MARTin. Microarrays are produced and processed and subsequently imaged. MARTin enables the digitalization of array signals across platforms including the adjustment for experimental errors such as spot/field offsets and background variability.

Here we present MARTin (Figure 1), an open-source software freely accessible on GitHub under the AGPL (Free Software Foundation, Inc, 2007). MARTin is a versatile and user-friendly array-analysis software, developed for the analysis of protein as well as peptide microarrays. Its features include background correction, a signal fitting algorithm, metadata management and compatibility across different microarray formats.

## 2 Implementation

### 2.1 Implementation and architecture

MARTin is built as a standalone application based on the image processing software ImageJ (Rueden et al., 2017), written in Java 11. While ImageJ itself is a powerful scientific image analysis software, out of the box it is not capable of analysing peptide microarray images efficiently. Thus, MARTin uses ImageJ as a software library to provide basic functionalities, such as the image viewer, quantification via regions of interest (ROI) and image filtering. MARTin is built on top of the ImageJ API and expands it greatly, allowing for a grid-like quantification of multiple signals at once, effective data management, enhanced ROI-positioning and *post hoc* measurement validation. The basic application is structured using the model–view–controller (MVC) architecture with a custom designed user interface (UI) using AWT/Swing. The look and feel (LaF) is provided through the excellent open-source FlatLaf (FormDev Software GmbH, 2023) project. During implementation, we made use of many functional paradigms to cut risks of introducing critical code bugs: for example, many classes are coded inherently immutable to additionally ensure correctness, in particular for the consistency of measurements. Additionally, we used unit testing to provide assurance over the post-measurement preliminary data analysis and algorithm validity. Finally, integration tests ensured consistent results over the version evolution and test overall vital business logic. Standard industry practices such as version control using Git, code review, and CI/CD were followed. We provide application installers for Windows and Linux distributions (RPM) using jpackage.

### 2.2 Mask positioning

**FIGURE 2:**
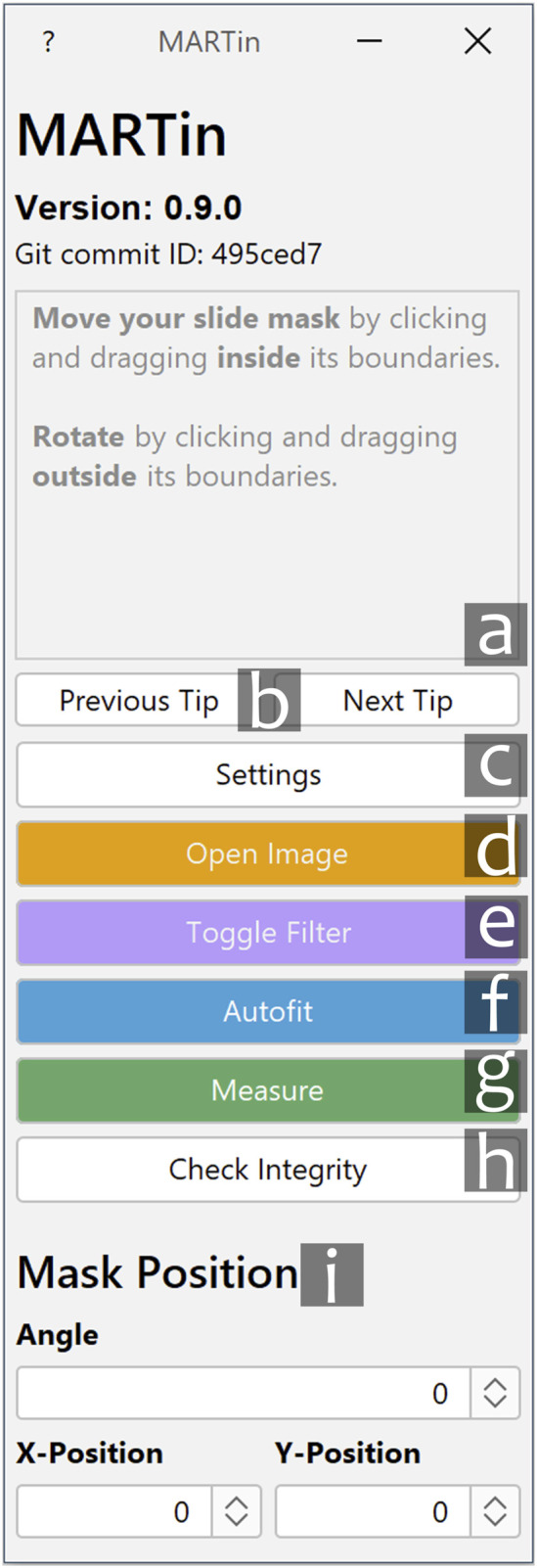
Main GUI of MARTin. a. Info text window. Displays useful tips and information for using MARTin. b. Cycles through different info messages. c. Settings menu. Opening the settings menu temporarily disables buttons c-g. d. Open Image. Opens a new image and closes all previously opened images. e. Toggle Filter. Toggles the adaptive filter for slide positioning. f. Autofit. Initiates the automatic fitting algorithm. g. Measure. Measures the content of the current slide mask and opens the export dialogue. h. Check Integrity. Validates the integrity of previously conducted measurements. i. Mask Position. Enables digital mask positioning. This allows for the manual input of the slide mask angle as well its horizontal (“X-Position”) and vertical (“Y-Position”) coordinates.

The main tool for the quantification of Microarrays in MARTin is called a “Slide Mask.” All measuring of a Slide Mask takes place within the “Measure Fields,” they, amongst the other elements of our Slide Masks, are explained in more detail in Section 3.3. Positioning of this mask is possible both analogously via mouse click-and-drag and digitally via the corresponding graphical user interface (GUI) elements, which are depicted in Figure 2.

### 2.3 Adaptive filter

To improve the manual positioning of a Slide Mask, adaptive filtering was added as a feature of MARTin. This filter utilizes local contrast normalization (LCN) which accounts for local peculiarities in the image and is independent of the overall brightness and differences between spots as well as varieties between assay manufacturers. The LCN uses a basic statistic block filter whereby the value of an output pixel is determined by the surrounding pixel block akin to a convolutional filter. Specifically, the kernel centres the pixel values around the mean as well as stretches the contrast within the block area specified. This is achieved by the following kernel formula:

$$\omega =\frac{I_{\text{range}}\cdot \left(v-\mu +\sigma \right)}{2\sigma }+I_{\text{min}}$$

Here, *I*_range_ corresponds to the maximum spread of pixel values over the whole image, e.g., *I*_max_ − *I*_min_. *v* is the current pixel value in the iteration. *μ* and *σ* are, respectively, the arithmetic mean and corrected sample standard deviation of the considered surrounding pixel block. The implementation of the LCN algorithm is provided through the “Integral Image Filters” plugin written by Saalfeld (Saalfeld, 2020). Our internal testing showed the best results with a block size of 40 pixels over the tested assays. The use of the adaptive filter for positioning has no influence on the measurement at all and is only displayed to the user.

### 2.4 Autofit algorithm

To effectively position each Measure Field at its local maximum, we devised a method that drew inspiration from the “Gradient Descent” algorithm. To securely find the absolute maximum for each cell, the step size of the algorithm was set to one pixel, ensuring the highest possible degree of accuracy. For each step of the algorithm, instead of single pixels, areas called “Search Fields” equivalent to a Measure Field (see Section 3.3) in size are checked. The main issue underlying this approach lies in the fact that the normal method for determining step sizes is not viable because, instead of a gradient, there is a sharp edge between signal and background on microarrays. However, this approach can still be used because the initial positions of the starting points for the “Gradient Descent” are chosen in such a way and number that a signal will always at least be partially covered. Because of a step size of one pixel and the tight population of our Search Fields, we can ensure that the local maximum will always be found, no matter the resolution of the microarray. To improve the running time of this algorithm adjustments were made to reduce redundant checks. This is done on the level of individual Search Fields and across all Search Fields of a Spot Field cell.

### 2.5 Microarray data quantification

Quantification includes the mean pixel value within a Measure Field, the maximum and minimum pixel value, as well as the sample standard deviation. Additionally, to create a dynamic range representation, the mean_minus_min value is calculated by subtracting the absolute minimum mean from each mean Measure Field value (Eq. 1). The normalization process involves dividing by the maximum mean_minus_min value, this is referred to as normalized_mean (Eq. 2). Furthermore, there is the option of aggregating between all grids used in a measurement. This means averaging between all duplicates and normalizing these values by the maximum. As a measurement of sameness between grid-copies, we also added the population standard deviation, in absolute and relative terms, for the mean and normalized values for each grid. This should only be used in cases where the grids are placed upon duplicates. This is a common quality control practice for Microarrays. The adjusted_average value field refers to the mean value between all mean_minus_min values of the same grid position (i.e., all values where row = 2 and col = 1). The meaning of the remaining values should be self-explanatory. If the Background Rectangles are enabled, their mean signal strength will be subtracted as noise for all pixels of an image before the actual measurement occurs. This will have no effect on the image itself; repeated measuring will not change it in any way. This is because all measuring takes place on a copied instance of the image, which is not visible to the user.

### 2.6 Workflow

The following is a comprehensive summary of the microarray-quantification workflow using MARTin, which will be explained in more detail in the following sections.

**FIGURE 3:**
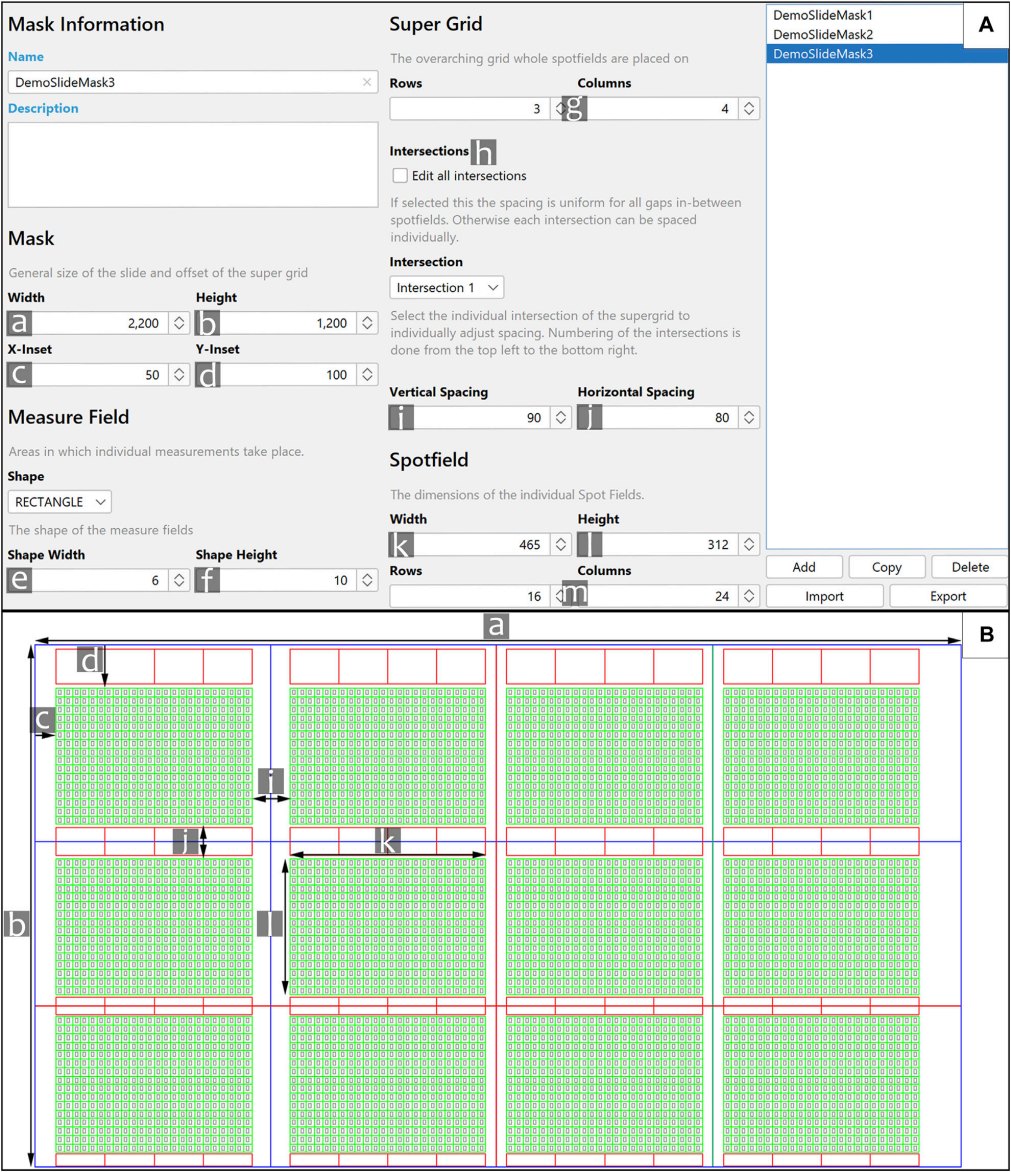
Slide Mask Designer and its application. **(A)** Shown is the Mask Designer. The parameters are marked with the letters a–m, they correspond to the same letters on **(B)**. e and f [not shown in **(B)**] allow to alter the size of the Measure Fields. g. The rows and columns of the super grid respectively represent the number of spot fields vertically and horizontally. h. Spacings between Spot Fields (see i and j) can be set the same for all spot fields (via “Edit all intersections”), or specifically for each spot field. Specific spacings can be selected via the corresponding intersection. This is depicted in **(B)**, the blue lines between Spot Fields are the first intersection; the red lines depict the second one. The colour of these lines were changed for demonstrative purposes. m. The number of rows/columns of all Spot Fields. **(B)** An example Slide Mask which is based on the inputs in **(A)**. The lines in blue depict the general outline of a slide. The red boxes above and below the slide are called “Background Rectangles.” The green grid is called a “Spot field.” The grey rectangles are the “Measure fields.”

1) Download the correct installer on the MARTin GitHub page (Kreissner and Faller, 2023a).
2) Double-click the installer and follow the install-guide.
3) Open up the MARTin software.
4) Open an image using the “Open Image”-button on the main GUI (Figure 2d).
5) Adjust the Slide-Mask to your assay by opening the settings menu (Figure 2c) and initiating the Slide Mask Designer (Figure 3).
6) Position your Slide-Mask on your microarray via click-and-drag or by using the “Mask Position”-sliders (Figure 2i).
7) Optional Steps:
  • Use the adaptive filter (Figure 2e) to simplify positioning (as depicted in Section 3.4).
  • Click the “Autofit”-button (Figure 2f) to automatically adjust the ROIs to the signals on your microarray.
  • Manually adjust specific elements of your slide mask via click and drag to fine-tune their positioning.
8) Click the “Measure”-button (Figure 2g).
9) Input the correct metadata in the export dialog (Figure 6B).
10) Click the green “Export”-button.
11) Select the path where you want to store your measurement and click “open”.

Of course the usual work routine starts at point three. Once a fitting slide mask has been created it can also be reused making point five unnecessary. Furthermore, point eight can be massively simplified by using projects, this is explained in further detail in Section 3.6. MARTin also provides the user with useful tips and information on its usage via the info text window on the main GUI (Figure 2a). Section 3.10 can also be seen as an example of this workflow in action.

## 3 Results and discussion

MARTin is geared towards compatibility with standard microarray formats as well as customs designs ranging from low-density to high density formats and including printed arrays with *x*- and *y*-offsets as well as laser-guided synthesized arrays. Once an image is imported into MARTin, the signals, which we call spots, can be quantified in a semi-automatic manner. They can then be exported as tab-separated values (TSV) and JavaScript Object Notation (JSON) files, which can be used for downstream data visualization in widely used software such as R Studio, Origin Pro, and GraphPad Prism. First, we describe and explain the use of MARTin and its features. Subsequently, the versatility and robustness of MARTin is highlighted through its application on six different arrays formats.

### 3.1 Main graphical user interface

This GUI allows the user to access all functions of MARTin. Function specific info-texts appear upon hovering over key elements of the interface. Specific features of the interface are explained in Figure 2.

### 3.2 Import of imaging data

Microarray images are loaded into MARTin using the “Open Image” (Figure 2d) button. In its current state MARTin is able to accurately measure single channel greyscale-, as well as RGB-images in a multitude of image formats, i.e., TIFF, PNG or JPEG. Multichannel images have to be split by their channels before measuring.

### 3.3 Application of the slide mask

**FIGURE 4:**
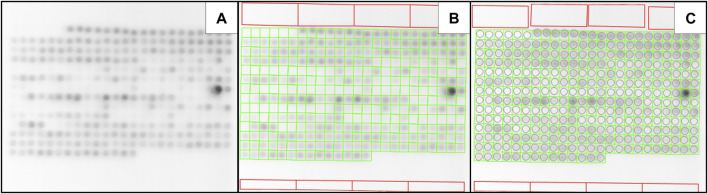
Adjusting a Slide Mask to fit deviating spots. **(A)** A microarray with vertical deviation in the first row of spots. **(B)** A slide mask was positioned over the microarray; the Measure Fields are set invisible for illustrative purposes. One can see that the deviation of the first couple of spots is so strong that they go beyond the bounds of the Measure Field. **(C)** Manually adjusted Slide Mask. Some spots were manually adjusted to capture the whole signal. The upper Background Rectangles were also moved away from the deviating spots.

After loading a microarray image into MARTin a Slide Mask needs to be defined and applied. The Mask Designer and a representative slide mask is shown in Figure 3. All measurements are done within the bounds of a “Measure Field.” These fields are polygons of circles, rectangles, or diamonds which are placed in the cells of a grid called a “Spot Field.” The user can set all dimensions of this grid, i.e., width, height, and the number of rows/columns. Multiple grids with the same dimensions can be freely generated next to one another. The spacing between these grids can also be altered by the user. The Spot Field serves as a help for general positioning and serves also a graphical depiction of the search boundary of our fitting algorithm. Additionally, there are toggleable “Background Rectangles.” These rectangular polygons are used to quantify and later subtract background noise. All of these elements are summarized as a Slide Mask, mirroring the physical composition of a Microarray. The Slide Mask is moved and rotated to roughly match the array format analogously via mouse-drag or digitally via the corresponding elements (Figure 2i). The manual pre-matching is facilitated through zooming in and out, by scrolling with the mouse wheel while the control-key is pressed. Additionally, if the space key is held down the user is able to move the whole image. The individual Measure Fields and Background Rectangles are also moveable via mouse-drag. This allows for manual adjustments in the case of an imperfectly produced sample, as can be seen in Figure 4. If the fitting algorithm is triggered all manual positioning will reset. The Mask Designer generates, stores, loads, imports, exports, and alters Slide Masks (Figure 3B). The combination of all features into one tool enables quick switching between multiple presets and sharing between users. Slide Masks can still be moved and rotated while the Slide Mask Designer is active. This enables the user to dynamically adjust the relevant parameters to easily produce a fitting mask. The number of active Measure Fields can also be selected separately via the settings menu. As such a Slide Mask only has to be adjusted for the general printing pattern of the slides and not for each separate project.

### 3.4 Adaptive filtering for rough positioning

**FIGURE 5:**
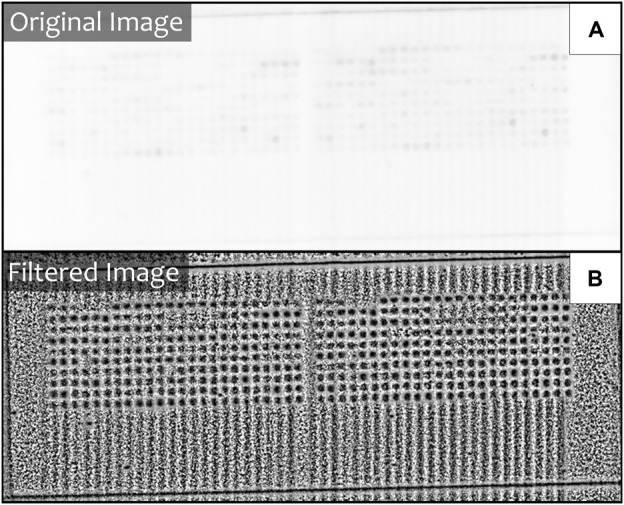
The effect of adaptive filtering. **(A)** A low signal image before the adaptive filter is activated. **(B)** The same image after the adaptive filter has been activated.

Measuring low-signal microarrays can pose a challenge, due to the associated difficulty of visually identifying spots to accurately position the mask. As such performing reliable quantification can prove to be difficult. Common solutions, such as increasing the contrast or adjusting the tonal range of the whole image, provide only an insufficient solution since they can easily shift outliers with a strong signal out of range without effectively enhancing the contrast between signal and background. Furthermore, those solutions require manual intervention due to their dependency on the overall brightness and image features. Instead, we provide a one button click solution (see Figure 2e) with our adaptive filter. Its main purpose is to make the whole grid of spots visible; this is depicted in Figure 5. Further technical details are described in Section 2.3.

### 3.5 Automated spot Correction—autofit algorithm

Slight deviations of spots occur naturally in the process of microarray printing. Larger *x* and *y* offsets are also created by the necessities to maintain solutions on the array which create torsion by introducing interfaces with different optical densities. As such a quantification software for microarrays has to be able to effectively handle these deviations. This is especially true for high density arrays with a very large number of spots, where manual corrections are not a viable alternative. In MARTin positioning of the Measure Fields over the actual signals is achieved by an automated fitting algorithm. By pressing “Autofit” in the main GUI (Figure 2f), each Measure Field will be placed at the position where the highest mean signal density, within the confines of its corresponding Spot Field cell, can be measured, i.e., its local maximum. Importantly, the applied algorithm ensures that the local maximum will always be found, independent of the resolution of the microarray. This is described in more detail in Section 2.4. In some cases, the deviation that occurs in printing may exceed the spot fields of a given Slide Mask. In these situations, we recommend manually positioning the Slide Mask to cover as many spots as possible, followed by using the autofit-algorithm, and making final adjustments manually by clicking and dragging the affected Measure Fields (as depicted in Figure 4).

### 3.6 Project Manager and metadata input

**FIGURE 6:**
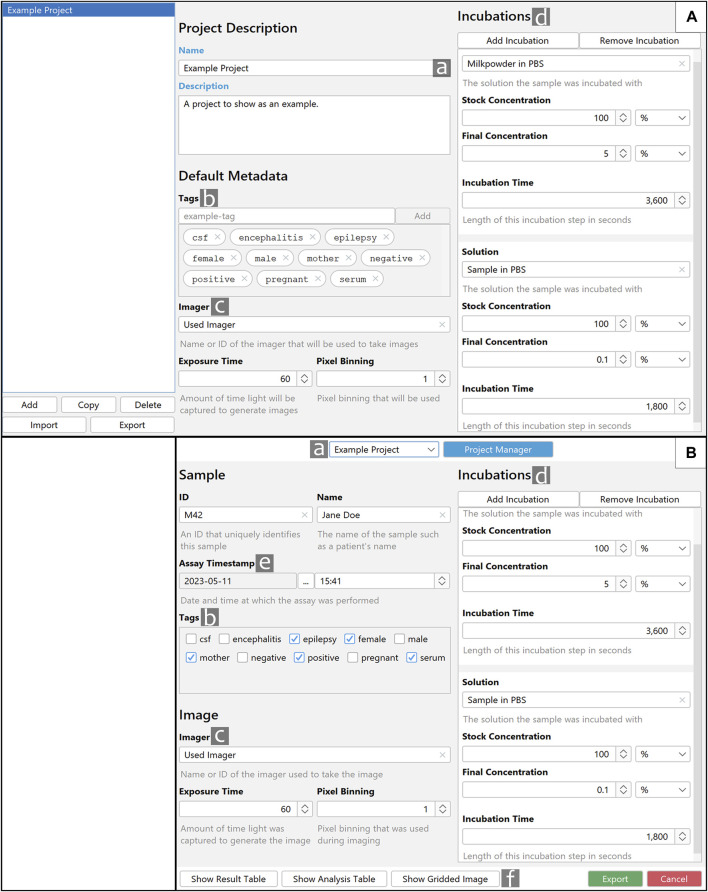
Layout and functions of the Project Manager and export dialogue. **(A)** Shown is the Project Manager with representative data. **(B)** Shown is an export dialogue which utilizes the “Example Project” created in **(A)**. a. The selectable project name. b. Tags that are defined in **(A)** can easily be selected in **(B)**. c and d. Predefined information is carried over from **(A)** to **(B)**. e. Assay Timestamp. If available, the timestamp reflects the image’s creation time; otherwise, the current time and date are used. f. The Buttons “Show Result Table,” “Show Analysis Table” and “Show Gridded Image” allow for a preview of the measuring results, in contrast the export button will store all data in a specified directory.

Before initiating a series of measurements, it is recommended to define its presets as a project via the Project Manager (Figure 6A) to ensure standardization. Metadata input is done via the export dialogue (Figure 6B), which is triggered for each individual measurement. The project structure allows for a quick and simple autofill for most of the metadata of an export. This is possible because the basic conditions of experiments within a set project are usually very similar to each other. For microarrays, this mainly applies to the number and content of the incubations performed. This also allows for an export of all files generated during a measurement, into an organized and easily readable folder structure. An automatic readout across a structure such as the one described can easily be accomplished. Furthermore, one of the biggest advantages of using projects is the tagging-function. Presetting a set of tags is mainly done to prevent semantic duplicates and ensure standardization. Tagging is a simple but powerful way to define different subsets of data, like, for example, positive-samples and negative-controls. If done correctly, this can be used to easily analyse across multiple datasets. To meet the current data management standards (Wilkinson et al., 2016), we strongly encourage the usage of projects in MARTin; this not only promotes a good scientific practice and improved reproducibility of microarray experiments but also ensures compliance with essential data management requirements.

### 3.7 Microarray data quantification and data export

Clicking on “Measure” (Figure 2g) will quantify the content within each of the Measure Fields and open the export dialogue. If a project is selected some parts of the Export GUI will be automatically filled with fitting metadata; this is depicted in Figure 6. All automatically filled values can still be changed manually if necessary. Tags can and should be selected for each individual measurement. Assay Timestamp will automatically be set to date and time the measured image was created. If this information is missing, the current time and date will be selected. An export consists of the data for the individual Spot Fields as well as aggregations between all spot fields. The specific values are explained in Section 2.5. Export formats and additional export content like an annotated image can be toggled in the settings menu.

### 3.8 Integrity check

Previous measurements can be validated with the built-in integrity check function of MARTin (Figure 2h). To use this function the user simply has to select a previously created export directory of a single measurement. MARTin then checks if the stored metadata, so the position and size of each element of a Slide Mask, can be used to recreate the measurement. The measurement integrity is considered valid if the replicated values fall within a margin of error of 10^–6^ of the original values. Manually altered values beyond this threshold are considered invalid. The integrity check does not prevent data manipulation but contributes to identify accidental alterations of microarray data sets. Additionally, if the export of an “Annotated Image” was enabled in the settings menu, each export will also have an image depicting the exact position of the Slide Mask and all its elements at the moment of measuring. This can be used to quickly check if the Slide Mask was positioned properly. The basic requirement for this function is that a copy of the initially measured image and the positional metadata of the measurement are both present in the selected folder.

### 3.9 Usability in different formats

**FIGURE 7:**
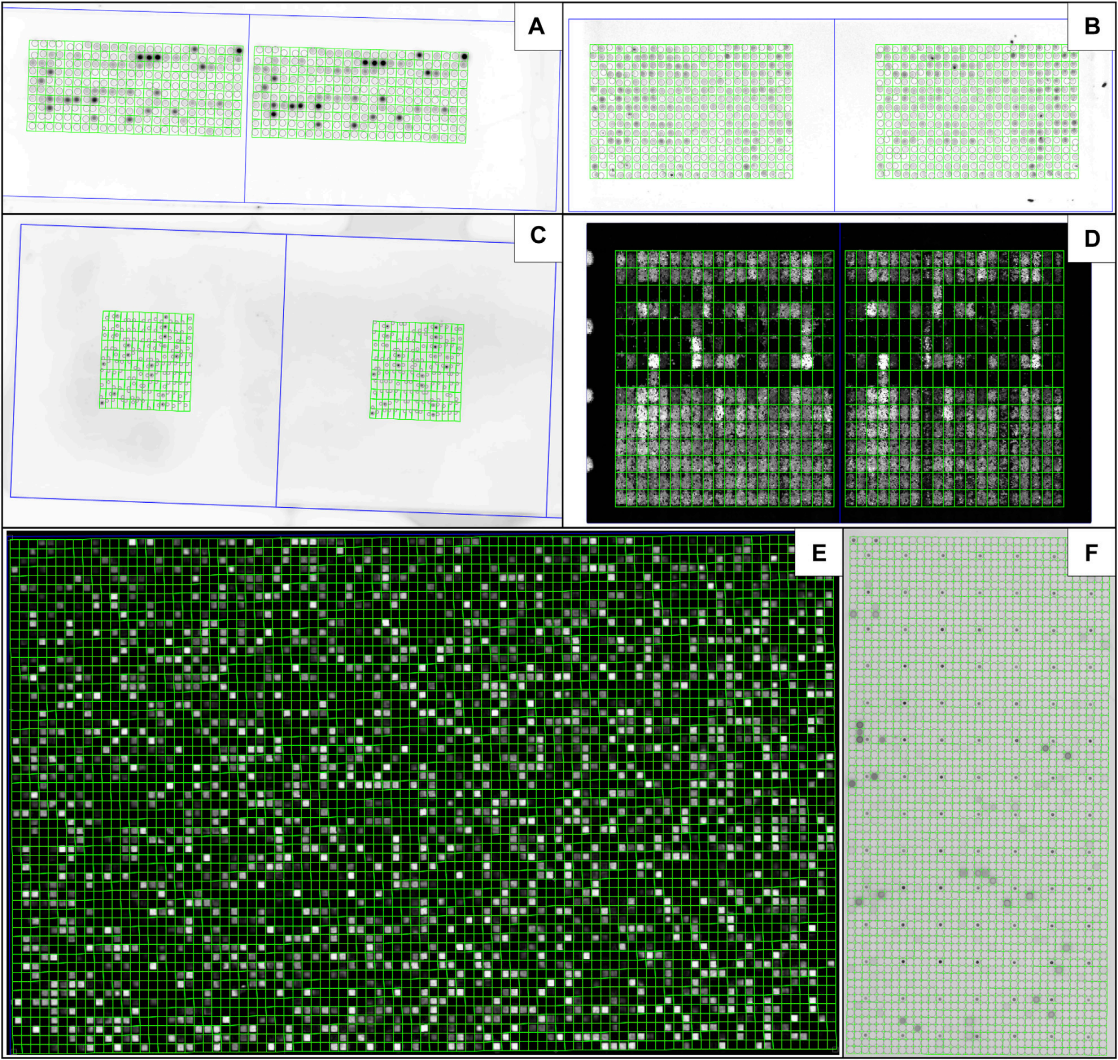
MARTin is adjustable to different microarray formats. Shown are in-house produced microarrays using the µSPOT principle (Schulte et al., 2023; Frank et al., 1992), using a contact printer (CEM GmbH) **(A)** or an Echo525 (Beckman Coulter) **(B)**, as well as high-density formats commercially available from JPT Peptide Technologies GmbH **(C)**, PepPerPrint GmbH **(D)**, Schafer-N **(E)** as well as Engine GmbH **(F)**. Background Rectangles were disabled for all masks. The Measure Fields were made invisible in **(E)** to ensure better visibility of the actual signals. **(A–E)** are peptide microarrays, **(F)** is a protein subarray.

The extensive versatility of MARTin is evident in its adaptability across diverse microarray formats, this is showcased in Figure 7. Notably Figure 7F shows the use of MARTin on a non-peptide microarray, in this specific case a protein microarray.

### 3.10 Variation in repeated measurements

Finally, we conducted repeated measurements of the same microarray with MARTin. This was mainly done to showcase the accuracy of our fitting algorithm, but it can also be viewed as a marker of measurement consistency and process speed (Table 1). The measurements were conducted by eight individuals over five distinct days, on each day the same microarray was measured ten times in succession. All participants were provided with an instructional video detailing the procedure. The measurements were carried out using the personal computers of the participants, in their respective home settings. Each positioning was done using the same mask, starting at the zero position, i.e., the top left corner of the image, with the Slide Mask set to zero degrees of rotation. The fitting algorithm was used for all measurements, no manual repositioning of individual spots was conducted, background subtraction was disabled and the adaptive filter was not used. In total 400 measurements were taken, we excluded eight of those measurements in our analysis because of significant mistakes made in the manual positioning of the Slide Mask. The individual measurements took on average 55.2 s (*SD* ± 16.4 s), while a whole measuring session of ten measurements took around 9 minutes. The average coefficient of variation across all spots was 4.6% for the minimum-, 1% for the maximum- and 0.9% for the mean pixel value on average over all participants. Eq. 3 shows the formula we used for the average coefficient of variation. The positional data, namely, the x- and y-coordinates, displayed on average a standard deviation of 0.6 pixels for x and 0.8 pixels for y. Eq. 4 shows the formula for the average standard deviation. Notably, six of our eight participants were first time users, some of which only showed a small gap in speed and measurement quality to our more experienced users (participants one and three). In contrast to the general average, our most experienced user (participant 1) took an average of 45.8 s (*SD* ± 9.3 s) for each measurement, displayed an average coefficient of variation of 2.7% for the minimum-, 0.4% for the maximum- and 0.4% for the mean pixel value and had an average a standard deviation of 0.4 and 0.5 pixels for the x- and y-coordinates respectively. The general variation between measurements of our participants can be depicted by calculating across all measurements instead of averaging between our participants. In this case the average coefficient of variation is 6.2% for the minimum-, 2.1% for the maximum- and 1.5% for the mean pixel value and the average standard deviation is 0.8 and 1.0 for the x and y-coordinates respectively. Table 1 gives an overview of our participants. The measurements were performed on an image consisting of 3326 × 2504 pixels, utilizing circular measure fields with a diameter of 14 pixels. Each measurement encompassed 480 individual Measure Fields. We rounded all values that were calculated for this section to the first decimal. All data used for this section, as well as the code we wrote for the statistical analysis is freely available on GitHub (Kreissner and Faller, 2023b). Any of the measurements done can easily be validated via our integrity check function (Section 3.8). We consider these findings to demonstrate only minimal degree of variation amongst measurements, thereby affirming the robust consistency of MARTin.

**TABLE 1:** Evaluation of MARTin analysis time and accuracy. Repeated measurement of the same microarray done by eight different people. Shown are the average and composite values for all eight participants as well as the overall average and the overall composite values. Participants are named P followed by their respective number (for example, P3 being participant three). The averages are generated by averaging the standard deviations of each measuring session. Composite values are generated by combining multiple sessions into one and calculating the standard deviation from this combined dataset. All values have been rounded to the first decimal.

	Min pixel value rel. StDev [%]	Max pixel value rel. StDev [%]	Mean pixel value rel. StDev [%]	X-coords stDev [px]	Y-coords stDev [px]	Duration average [sec]	Duration StDev [sec]
Session average							
P1	2.7	0.4	0.4	0.4	0.5	45.8	9.3
P2	3.6	0.5	0.7	0.6	0.6	46.0	12.0
P3	2.8	0.4	0.5	0.5	0.6	49.2	10.7
P4	7.6	2.1	1.8	0.9	1.3	38.2	9.6
P5	7	1.8	1.6	1.0	1.1	34.5	10.3
P6	2.5	0.3	0.3	0.4	0.5	106.3	46.3
P7	4.6	0.6	0.8	0.6	0.6	71.0	24.5
P8	6.3	1.4	1.3	0.8	0.9	50.8	8.6
Composite sessions							
P1	3.4	0.5	0.5	0.5	0.5	45.8	5.8
P2	4.4	0.7	0.8	0.6	0.7	46.0	7.7
P3	3.5	0.6	0.6	0.5	0.6	49.2	3.3
P4	8.3	2.9	2.1	1.0	1.4	38.2	6.5
P5	8.2	2.8	2.0	1.2	1.3	34.5	9.2
P6	3.1	0.5	0.5	0.4	0.5	106.3	33.0
P7	5.3	0.8	1.0	0.6	0.7	71.0	28.0
P8	7.5	2.2	1.7	0.8	1.0	50.8	12.8
Overall							
Average	4.6	1.0	0.9	0.6	0.8	55.2	16.4
Composite	6.3	2.1	1.5	0.8	1.0	55.2	26.9

## 4 Conclusion

Here we introduce MARTin as a fast and robust solution for microarray quantification. In contrast to tailored commercial solutions MARTin is compatible to a variety of different microarray formats and allows for accurate quantifications independent of the imager or sample-printer used. Crucially, this allows the comparison of results not only across various experiments but also between different platforms and even array types. It can be expected that this will improve reproducibility and meta studies including overlapping array data sets. Furthermore, the project-oriented structure of MARTin serves as an easily accessible way to document assays with emphasis on metadata management and good scientific practice. To this end, the structured data-export also provides an interface ready for additional subsequent data processing such as tagging, export, statistical analysis, and direct visualization.

It is important to note that MARTin is a relatively new software and therefore not extensively tried and tested for all of its possible applications. However, over the course of its development process, it underwent multiple rounds of testing and fine-tuning, directly accommodating to the input of our users. In fact, it has already been routinely deployed in real-life applications by its community of beta-testers.

It is also noteworthy that the majority of the microarrays showcased in this publication are peptide microarrays. This is because MARTin was primarily developed for and tested on this specific type of array. However, the primary challenge in quantifying microarrays lies not in the sample type, but in variations of the printing format. In abstract terms, MARTin quantifies the signal strength of spots within a grid. As long as a grid-based format is employed, it is feasible to quantify it using our software thanks to its high adaptability (see Section 3.9).

One of the primary motivations behind the development of MARTin was the absence of a universally recognized gold standard in microarray quantification. The fact that many of the most prominent alternatives in this field come with commercial price tags posed an additional obstacle. In contrast, MARTin’s open accessibility allows for independent testing, enabling potential users to make an informed choice if they want to use MARTin for microarray quantification. These factors collectively informed our decision to refrain from direct comparisons with other software in this publication.

Furthermore, there is still potential for enhancing the MARTin framework. For instance, implementing a fully automated detection and precise fitting of Slide Masks to microarrays would be feasible with the aid of positional markers on the arrays themselves. This advancement would significantly amplify the efficiency of MARTin and unlock its potential for deployment in high-throughput scenarios. Additionally, incorporating further features into MARTin would expand its applicability across various use-cases, offering ample opportunities for future improvements and the expansion of our software. In summary, we have shown that MARTin is a powerful and user-friendly software that can be widely used for the accurate quantification of microarrays for a large variety of assays. As such MARTin could serve as a first step towards building a reference database which is currently missing for proteomic microarray data.

## 5 Availability and requirements

**Project name:** MicroArray Rastering Tool—MARTin

**Project home page:** https://github.com/scitequest/martin

**Archived version:** Version v0.11.0 with Git commit ID: b5b00bffcf6d864b2b9d6c6077010554c9934b98

**Operating system(s):** Platform independent (Binaries as RPM and EXE)

**Programming language:** Java

**Other requirements:** Java 11 (and Java 17+ to build installers)

**Licence:** AGPL

## 6 Formulae

$$\mu_{\text{adjusted}}=\mu_{i}-\mu_{\text{min}}$$ (1)

*μ*_adjusted_ is the adjusted mean.*μ*_*i*_ represents the *i*-th value in a set of averages.*μ*_min_ represents the minimum average in the set.

$$\mu_{\text{normalized}}=\frac{\mu_{i}-\mu_{\text{min}}}{\mu_{\text{max}}-\mu_{\text{min}}}$$ (2)

*μ*_normalized_ normalized adjusted average value.*μ*_*i*_ represents the *i*-th average in the set.*μ*_min_ represents the minimum average in the set.*μ*_max_ represents the maximum average in the set.

$$\bar{c_{v}}=\frac{1}{m}\overset{m}{\underset{i=1}{\sum }}\frac{\sigma_{i}}{\mu_{i}}$$ (3)

$\bar{c_{v}}$ average of a set of relative standard deviations.*m* denotes the number of measurements.*σ*_*i*_ represents the population standard deviation for the *i*-th index across all measurements.*μ*_*i*_ represents the population mean for the *i*-th index across all measurements.

$$\bar{\sigma }=\frac{1}{m}\overset{m}{\underset{i=1}{\sum }}\sigma_{i}$$ (4)

$\bar{\sigma }$ average of a set of standard deviations.*m* denotes the number of measurements.*σ*_*i*_ represents the standard deviation for the *i*-th index across all measurements.

## Data Availability

The datasets presented in this study can be found in online repositories. The names of the repository/repositories and accession number(s) can be found in the article/supplementary material.
